# Early-life enrichment in American mink (*Neogale vison*): Effects of juvenile physical enrichment on behaviour, temperament, and long-term stereotypic behaviour

**DOI:** 10.1017/awf.2024.72

**Published:** 2025-01-13

**Authors:** Gabrielle B. Clark, María Díez-León, Rebecca K. Meagher

**Affiliations:** 1 Dalhousie University Faculty of Agriculture, Department of Animal Science and Aquaculture, Truro, Nova Scotia, Canada; 2 Royal Veterinary College University of London, Department of Pathobiology and Population Sciences, London, UK

**Keywords:** American mink, animal welfare, early-life environment, environmental enrichment, stereotypic behaviour, temperament

## Abstract

A single manipulable enrichment is often introduced to the pens of farmed American mink (*Neogale vison*) to combat stereotypic behaviour and behaviours or temperaments associated with poor welfare (e.g. inactivity, fear, and aggression). This enrichment is provided early in life, but it is unclear the age at which enrichment is most effective at preventing stereotypic behaviour and ameliorating welfare. Here, a group of enriched kits (EK) were provided with multiple enrichments that were periodically exchanged to renew novelty from 4–15 weeks of age, earlier than typical enrichment provision on farms, after which they were housed with a single standard enrichment into adulthood. The effects of EK enrichment on kit behaviours and long-term stereotypic behaviour were compared to that of two groups reared with a single standard enrichment (standard housed; SH and enriched at whelping; EW). Inactivity in the nest-box was decreased in EK kits as juveniles relative to other groups, however, social play was reduced and lying awake was increased compared to EW and SH juveniles, respectively. Stereotypic behaviour in the kits as adults was not prevented by EK interventions; in fact, EK kits may have developed more diverse sub-types of stereotypic behaviour than EW and SH kits. Moreover, kit temperament did not appear to be affected. EK enrichment may have been ineffective in improving welfare due to the timing of its removal or potential frustration induced by its removal. Recommendations are provided for future research regarding critical periods of enrichment to improve welfare in species such as farmed mink.

## Introduction

In order to promote the expression of species-specific behaviours and improve welfare in farmed furbearers such as American mink (*Neogale vison*), environmental enrichment (EE; defined in welfare science as changes or additions to animals’ environments or husbandry that are biologically relevant to the species; Newberry 1995) is often incorporated in pens. As per the most recent mink farming guidelines in Canada (National Farm Animal Care Council 2013) and the United States (Fur Commission USA 2019), one manipulable EE must be provided in each pen; mink in Europe were required to be housed with EE from 2008 onwards, although records show that 88% of mink in Norway were already provided with ‘activity objects’ by 2001 (European Commission 2001). The inclusion of manipulable EEs in mink pens has been found to prevent or reduce stereotypic behaviour, physiological stress, self-harm behaviours like tail chewing, and negative affective states such as fear or boredom (e.g. Hansen & Jeppesen 2000; Hansen *et al.* 2007; Dallaire *et al.* 2011; Meagher & Mason 2012; Díez-León *et al.* 2013, 2016; Meagher *et al.* 2014). Manipulable EEs may also have applications for modulating mink temperament: enriched mink have shown reduced fear and aggression in temperament tests and handling scenarios, sometimes paired with increases in explorative or curious responding (Meagher *et al.* 2014; Bak & Malmkvist 2020).

The EE provided is typically a mobile object within the pen that mink can chase and chew, which creates an outlet for innate behaviours (e.g. hunting) that are otherwise restricted in captivity and reduces underlying behavioural frustrations (Maple & Perdue 2013). The object(s) may target stereotypic behaviour by reducing stress or arousal arising from aversive environments, offering greater opportunity to exert control over the environment, and/or by occupying the animal’s time with other behaviours (Mason *et al.* 2007). Stereotypic behaviour may alternatively arise from central nervous system dysfunction or neuroanatomical changes occurring after time spent in captivity, namely in reward-sensitive areas or areas responsible for inhibiting repetitive, habit-like behaviours, in which case EE can be used to protect against these changes (Díez-León *et al.* 2019; Tatemoto *et al.* 2022).

The ontogeny of stereotypic behaviours in captive animals remains poorly understood, but they are estimated to become fully developed by seven months of age in mink (Jeppesen *et al.* 2000). EE is relatively unsuccessful at reducing stereotypic behaviour if provided after animals have reached maturity (Mason 1993; Ahola *et al.* 2011; Campbell *et al.* 2013), and animals can show novelty-induced fear responses or reduced motivation to gain access to enrichments when introduced to them as adults (Cooper *et al.* 1996; Tilly *et al.* 2010; Fairhurst *et al.* 2011). It is therefore recommended that EE for farmed mink be provided early in life. Although these practices have contributed to partial improvements in the occurrence of self-mutilation behaviours (e.g. tail chewing), stereotypic behaviour remains widespread (current prevalence is unknown but was estimated to occur in 35–85% of adult females based on a large-scale Netherlands study; reported by the European Commission 2001). Moreover, there is no mandated age of EE introduction on commercial mink farms in Canada, and optimal durations and/or timing of EE provision have not yet been identified. A recent assessment of mink welfare across fur farms in Europe assigned the lowest scores to the category of ‘Appropriate Behaviour’, which evaluates stereotypic behaviour, fur chewing, and temperament as well as cage enrichment (Henriksen *et al.* 2022), lending further support to the sub-optimal effectiveness of current enrichment practices on fur farms.

The present study is part of a larger experiment (i.e. on the same farm using the same cohort of mink) with several objectives relating to enrichment of the physical environments of farmed mink at different life stages. In the companion article to this study published at the same time (Clark *et al.* 2025), we presented the effects of enriching the perinatal environment with manipulable enrichments and premium bedding materials for pregnant dams compared to housing dams in standard conditions. Here, we aimed to compare the effects of such enrichment to that of supplying mink in a different group with above-standard EE in the early juvenile phase (once kits become mobile). Specifically, we aimed to investigate whether physically enriching the juvenile environment of farmed mink can positively modulate behaviour, temperament, and long-term stereotypic behaviour compared to mink housed with standard enrichment as juveniles. It was hypothesised that enriched kits would demonstrate a reduction in behaviours and temperaments associated with poor welfare (e.g. boredom, stress, fear, and aggression) and reduced stereotypic behaviour development later in life due to the greater behavioural opportunities available. It was demonstrated in a previous study, also based on this cohort of mink (Clark *et al.* 2023), that the variety and novelty of EEs provided to the enriched kits were successful at increasing and sustaining their use of enrichments compared to kits housed in standard conditions; thus, the juvenile enrichment condition was predicted to deliver a positive impact on behaviour and welfare.

## Materials and methods

### Subjects and housing

Male and female mink selected at breeding for potential use in the study were balanced across Dark, Mahogany, Pastel, Demi, and Stardust colour types (strains). Selection was carried out in advance because one treatment (EW) began before whelping, but we needed to account for later exclusions due to potential unsuccessful copulations and/or poor litter health. All dams were housed individually (American mink are solitary in the wild; Dunstone 1993) indoors at the Canadian Centre for Fur Animal Research (Nova Scotia, Canada) in 75 × 30 × 45 or 40 cm (length × width × height) wire-mesh pens with a wire shelf (25 × 30 × 25 cm), external wooden nest-boxes (25 × 30 × 20 or 18 cm), and a single plastic ring enrichment (3.8 cm thick, 10 cm in diameter) prior to assignment of their respective conditions. Mink were fed with a meat-based paste placed on the mesh roof of the pen; feedings were once daily in the afternoon for non-reproductive adults and twice daily (morning and afternoon) for pregnant dams approaching parturition, lactating dams, and kits. All mink had *ad libitum* access to drinking water via automatic drinkers. The research was approved by the Dalhousie University Faculty of Agriculture Animal Care & Use Committee (#1033575) and the Clinical Research Ethics Review Board of the Royal Veterinary College (URN 2021 2034-3).

#### Housing in the peri-whelping period

Dams who bred successfully (n = 242) were randomly assigned to one of three experimental groups: standard housing (SH; n = 59), enriched at whelping (EW; n = 119; relevant to Clark *et al.* 2025), or enriched kits (EK; n = 64). Groups were balanced for colour type and parity and pens were evenly distributed throughout the barn to account for potential effects of variable lighting, temperatures, noise levels, etc. The conditions for the EW group are described in the companion article to this study (see Clark *et al.* 2025), but in brief, dams assigned to the SH and EK housing conditions were given standard nest-building materials (chopped straw with wood chip bedding) while EW dams were given standard nest-building materials in addition to two ‘premium’ nest-building materials and a hanging sisal rope enrichment. These EW enrichments were in effect until post-whelping day 8.

**Figure 1.**
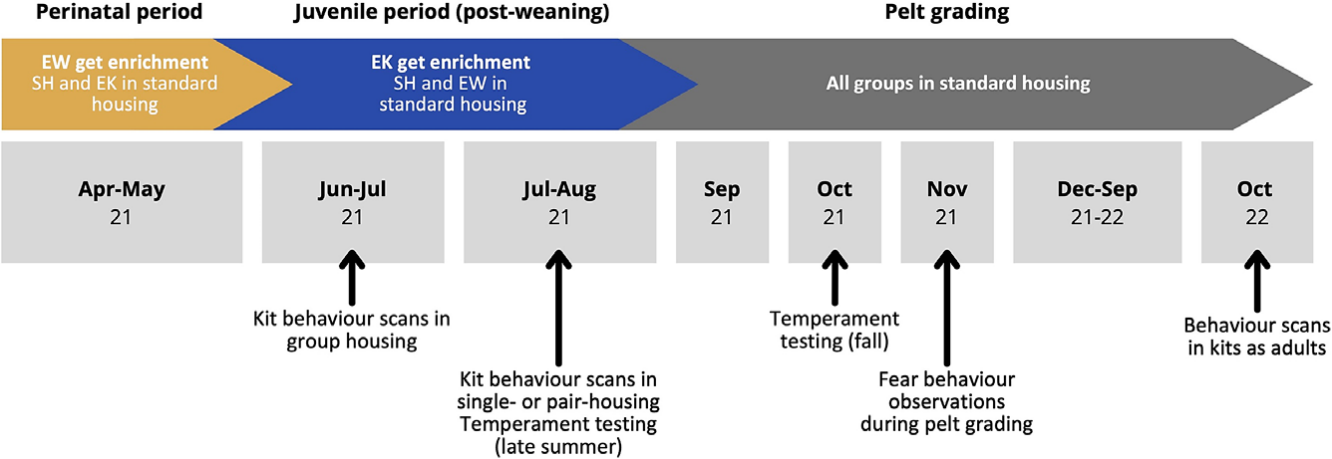
Timeline of standard mink-farming events taking place during the study (top), interventions for experimental groups (middle), and data collection for various tests (bottom). Months are indicated in grey boxes with the year (‘21’ denoting 2021 or ‘22’ denoting 2022).

When kits were approximately four weeks of age (28 [± 7] days post-whelping, when kits were expected to become mobile; Jonasen 1987), kits assigned to the EK condition were provided with a hanging plastic chain approximately 38–43 cm in length depending on cage height (selected based on beneficial effects demonstrated in a previous study; Meagher *et al.* 2014) in addition to a standard plastic ring enrichment (previously described). Dams and litters in SH and EW had continued access to a standard plastic ring enrichment at this stage (for a timeline of housing interventions for each group, see Figure 1). Litters were excluded from further testing if fewer than four kits survived to this point.

#### Housing in the post-weaning period: Group housing

Kits were weaned at six weeks of age as per standard farm protocols, at which point the dam was removed from the whelping pen and housed in a separate standard pen (number of litters surviving to this stage after exclusions: n_SH_, n_EK_, and n_EW_ = 36, 33, and 47, respectively). Kits remained in the whelping pen and were housed in groups of four to six (n_SH_, n_EK_, and n_EW_ = 218, 183, and 207 kits, respectively); if there were more than eight kits in a litter, they were split across two pens (applied to ten SH pens, three EW pens, and seven EK pens). Enrichment provision for EK kits through this period is detailed in Clark *et al.* (2023), but in brief, access to the hanging chain and standard EE was maintained in addition to introduction of a second mobile enrichment (wiffle ball or golf ball); enrichments were chosen based on beneficial effects demonstrated in previous studies (Díez-León *et al.* 2013; Meagher *et al.* 2014; Díez-León & Mason 2016). A schedule of enrichment exchange was implemented for EK kits such that mobile EEs were exchanged bi-weekly and hanging EEs were exchanged monthly to maintain object novelty (see Clark *et al.* 2023). Access to a standard ring enrichment was maintained for SH and EW kits with no enrichment exchange.

#### Housing in the post-weaning period: Pair housing

At ten weeks of age (four weeks post-weaning), kits were moved to single- or pair-housing pens according to standard farm protocol. One male and one female from each litter were chosen for pair housing and remained in the whelping pen (dimensions of 75 × 30 × 45 or 40 cm [length × width × height]; n_SH_, n_EK_, and n_EW_ = 46, 37, and 42 pairs, respectively). A single female from each litter was moved to a drop-in cage (a cage with a wooden nest-box connected at the back near the ceiling, such that mink have to jump up into them; dimensions of 76 × 25 × 45 cm; n_SH_, n_EK_, and n_EW_ = 21, 23, and 27 females, respectively). Male-female pairs and single-housed females in EK continued to have access to rotating enrichments (a standard ring, a hanging EE now in the form of a hanging sisal rope, and a second mobile EE now in the form of a pig’s ear or hockey ball; for detailed methods, see Clark *et al.* 2023) until 15 weeks of age, at which point these were removed and only a standard enrichment remained. Male-female pairs and single-housed females in SH and EW maintained access to a standard ring enrichment throughout this period.

### Kit behaviour observations

#### Kit behaviour scans in group housing

Following weaning, instantaneous scan-sampling observations of all pens were conducted three consecutive days per week for the following four weeks (from weaning to 10 weeks of age; constituting 12 days of observation per subject with multiple scans per day) by one experienced observer and two trained observers (note: observers were not blind to housing conditions or hypotheses being tested). Inter-rater reliability was assessed using Cohen’s kappa before observers began conducting observations independently (minimum score of 0.61–0.80; for scores in the moderate reliability range [e.g. a score of 0.57 for EE use], discrepancies were reviewed to improve reliability going forward). Scan-sampling observation methods are detailed elsewhere (Clark *et al.* 2023). Kits’ interactions with enrichments were recorded in addition to social play and resting (serving as ‘good’ welfare behaviours), aggression, defensiveness, lying awake, or general inactivity (serving as ‘poor’ welfare behaviours; locations in the pen were noted for the latter two behaviours to assess degree of ‘hiding’ in nest-box; Meagher *et al.* 2013), and activity, which served as a control behaviour (for ethogram, see Table 1). Pen observation order was reversed each scoring day to prevent the systematic scoring of certain pens earlier than others.

**Table 1.** Ethogram for adult and kit behaviour scans in farmed American mink (*Neogale vison*)

Behaviour		Description
Adults only	Stereotypic behaviour (SB)	Locomotor SB – Movement of whole body including directed movement of hind legs; translocation (e.g. the mink pacing back and forth within the pen). Performed as three or more consecutive repetitions (adapted from Díez-León *et al.* 2016; Polanco *et al.* 2017).
		Whole-body SB – Movement of upper body with no directed movement of hind legs; stationary, e.g. the mink moving the upper body from side to side (‘weaving’) or up and down the pen wall (‘twirling’). Performed as three or more consecutive repetitions (adapted from Polanco *et al.* 2017, 2018).
		Head-based SB – Movement of head only, e.g. the mink moving the head from side-to-side (‘head weaving’) or up and down the pen wall (‘head twirling’). Performed as three or more consecutive repetitions (adapted from Polanco *et al.* 2017, 2018).
		Wire gnawing – Chewing bars of pen. Persists for at least 3 s.
		Scrabbling – Digging at nest box bedding or pen floors/walls. Both forepaws must be engaged in the digging motion. Persists for at least 3 s (adapted from Meagher *et al.* 2013).
	Borderline SB	Movement pattern interrupted before three repetitions, or switching occurs between elements of common stereotypies without repeating a sequence three times. Not analysed as stereotypies but instead included in overall activity (e.g. Díez-León *et al.* 2016).
Kits only	Social play	Biting, pushing with nose, hitting with forepaws, chasing, pouncing on, or wrestling with another mink, without signs of aggression (see below; Dallaire & Mason 2016).
	Aggression	Resembles social play but with audible hissing, screaming and/or persistent escape attempts by one mink (Dallaire & Mason 2016); behaviour code is assigned to perpetrating member of the interaction.
	Defensiveness	As above, but behaviour is assigned to non-perpetrating member of the interaction (i.e. hissing, screaming, or attempting to escape).
	Enrichment use	Head in contact, licking, or sniffing enrichment within 1 cm; excludes sleeping with the enrichment.
All ages	Resting	Inactivity with head down and eyes closed or hidden (adapted from Meagher *et al.* 2013).
	Lying awake	Inactivity with eyes open (adapted from Meagher *et al.* 2013).
	Inactivity	Lying motionless other than slight postural adjustments; category used if observer can’t tell if resting vs lying awake (adapted from Meagher *et al.* 2013).
	General activity	Engaged in activity not in any of the above categories; includes eating, drinking and grooming self.

#### Kit behaviour scans in single or pair housing

All male-female pairs remaining in the study (n_SH_, n_EW_, and n_EK_ = 55, 43, and 44 pairs, respectively) continued to be observed according to the group-housing observation protocol until 13 weeks post-whelping. Females placed in single housing were not observed at this time, but EE rotation for EK females continued.

### Behavioural observations in kits as adults

#### Temperament testing

Following the juvenile observation period (and after additional EEs had been removed from EK pens), all single- and pair-housed kits remaining in the trial were tested for temperament by two trained observers using the stick test; both observers were blind to the previous housing conditions of the kits. This test involves inserting a popsicle stick into the pen and recording kits’ behavioural responses (Meagher *et al.* 2011; Mononen *et al.* 2012). Reaction categories included ‘curious’, ‘aggressive’, ‘fearful’, or ‘unresponsive’ (for ethogram, see Table 2). Tests were repeated over two consecutive days to determine the reliability of temperament scores for each subject (i.e. whether kits’ responses to the stick test were consistent across tests), and two rounds were conducted per day to revisit any kits who were sleeping in the first round. Kits with conflicting responses across testing days were excluded from analysis; responses were considered conflicting if kits responded as ‘curious’ on the first test and ‘fearful’ or ‘aggressive’ on the second test (Meagher *et al.* 2011). However, a ‘fearful’ reaction followed by a ‘curious’ reaction, an ‘aggressive’ reaction followed by a ‘curious’ reaction, or a ‘curious’ reaction followed by an ‘unresponsive’ reaction were accepted, as some decrease in fear/aggression or interest in the stick due to habituation was expected over repeated tests. In these cases, only the subject’s initial response was kept. Stick testing was conducted in the late summer when kits were 14–16 weeks old (n_SH_, n_EK_, and n_EW_ = 113, 101, and 107 kits, respectively) and repeated in the fall at 27–29 weeks (n_SH_, n_EK_, and n_EW_ = 111, 100, and 106 kits, respectively; some kits were lost between tests due to mortality or morbidity) to account for potential changes in kit temperament with age.

**Table 2.** Stick test response categories for temperament testing in farmed American mink (*Neogale vison*) (modified from Meagher *et al.* 2011)

Score	Description
Fearful (F)	The mink moves away from the stick or, if initially standing as far from the stimulus as possible, remains at that distance while attending to the stimulus for at least 30 s
Curious (C)	The mink approaches and sniffs the stimulus; they can make tooth contact without a hard bite, i.e. without closing their teeth - a gentle, exploratory nibble
Aggressive (A)	The mink rapidly delivers a hard and sustained bite (clamping their teeth together fully) to the stimulus
Unresponsive/ other (N)	The mink is alert and faces the stimulus but does not respond in one of the specified ways.

#### Fear behaviour observations during pelt grading

As part of standard farm processes, pelt grading was conducted in the late fall (30–32 weeks of age). All pair- and single-housed trial kits were captured and restrained on a pelt grading table to assess their pelt quality. During this process, the number of discrete vocalisations emitted by mink were counted and other behavioural instances including physical struggling, attempts to bite the handler, and urination were recorded using 1–0 sampling as additional measures of temperament and fear behaviour (Zieliński *et al.* 2019).

#### Behaviour scans in kits as adults

One year following the conclusion of juvenile observations and the removal of additional EEs for the EK group (at approximately 16 months of age), stereotypic behaviour (SB) of various subtypes (i.e. locomotor, whole-body stationary, head-based, scrabbling, and wire-gnawing; see Table 1) were scored in the remaining females on trial (n_SH_, n_EK_, and n_EW_ = 31, 37, and 36, respectively). Behaviours such as activity, resting, lying awake, or inactivity, and relevant locations of the latter two behaviours in the pen (Meagher *et al.* 2013), were also recorded (see Table 1). All mink were single housed by this stage, with access to a single standard ring enrichment. Instantaneous scan-sampling observations were conducted by an experienced observer with previous training in mink behaviour over five non-consecutive days in a two-week period from October to November (note: locations of inactivity sub-types in the pen were only noted during the latter three days of analysis). The observer was blinded to the females’ previous housing conditions. Pens were observed four times per day for a total of 20 scans per female; all rounds were conducted between 1200 and 1500h (morning feeding occurred at 0800h and afternoon feedings were at approximately 1500h). The behaviour being exhibited by mink upon observation was recorded before moving to the next pen, with a 30-s habituation period if necessary (i.e. if mink appeared vigilant of the observer and/or if a behaviour could not be classified without prolonged observation, such as a stereotypic behaviour that must be repeated a certain number of times). One round of sampling took 30 (± 15) min, thus instantaneous scans for each female were approximately 30 mins apart.

### Statistical analysis

All statistical analyses were conducted with jamovi statistical software (the jamovi project, 2023; v 2.3.18.0 for Mac). Figures were generated using Prism (GraphPad Software, 2023; v 10.02 for Mac). Significance level was set at *P* < 0.05. Assumptions of normality and homogeneity of variances for parametric analyses were assessed using Shapiro-Wilk and Levene’s tests, respectively. Transformations were performed as necessary (either square-root or log_10_ transformations, as appropriate) with mean and 95% confidence interval (CI) subsequently back-transformed for presentation. Where parametric analyses were not appropriate, non-parametric alternatives were used.

#### Analysis of housing effects on kit behaviours as juveniles

Behavioural scan data pertaining to kit activity, social play, aggression, resting, inactivity, and lying awake were formatted for analysis by calculating the average proportion of observations where a behaviour occurred based on the total number of observations (see equation below). This calculation was necessary because kits did not all receive the same number of scoring days due to scheduling constraints among the researchers and farm staff (as a result of farm events, etc).

Average proportion of observations where behaviour occurred:

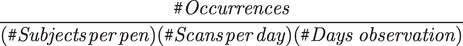

Average proportions of observations where behaviours occurred were analysed using one-way ANOVA (Welch’s, to account for unequal variances) and Tukey’s *post hoc* comparisons with housing condition as a factor when assumptions of normality and homogeneity of variances were met. Kit sex or colour type were not included as factors in these parametric analyses as there is little precedent in the mink literature to suggest that the behaviours measured vary between colour types or sexes at this age. Where data were not normally distributed and transformations were not successful, analyses were conducted using non-parametric one-way ANOVA and Dwass-Steel-Critchlow-Flinger pair-wise comparisons.

#### Analysis of housing effects on kit temperament as adults

Counts of temperament categorisations from summer and fall stick tests were compared across pair-housed kits and single-housed female kits using separate Chi-squared tests of association, with housing condition across rows and response categories across columns. Fisher’s exact test was used as needed to account for cells with counts below five. The same method was applied for occurrences (yes/no) of attempts to bite the handler, physical struggling, and urination during pelt grading. Number of vocalisations emitted by pair- and single-housed kits across housing conditions were compared using non-parametric Kruskal-Wallis one-way ANOVA.

#### Analysis of housing effects on kit behaviours as adults

Behavioural scan data pertaining to female kits’ stereotypic behaviour performance, resting, lying awake, or inactivity as adults, as well as locations of inactivity subtypes, were formatted for analysis by calculating the average proportion of observations where a behaviour occurred based on the total number of observations (see equation below).

Average proportion of observations where behaviour occurred:

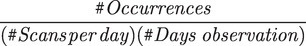

Average proportions of observations where behaviours occurred were analysed using one-way ANOVA (Welch’s, to account for unequal variances) and Tukey’s *post hoc* comparisons with housing condition as a factor when assumptions of normality and homogeneity of variances were met. Where data were not normally distributed and transformations were not successful, analyses were conducted using non-parametric one-way ANOVA and Dwass-Steel-Critchlow-Flinger pair-wise comparisons.

## Results

### Housing effects on kit behaviours as juveniles

#### Group housing

There was no effect of housing on any measures of kit behaviour in the group-housing phase (for a summary of statistical results, see Table 3[a]).

**Table 3(a).** Effect of housing condition (standard housed, SH; enriched kits, EK; or enriched at whelping, EW) on proportions of observations where juvenile behaviours in farmed American mink (*Neogale vison*) were observed in the group-housing period. SEM is given for measures analysed parametrically and SD is given for measures analysed non-parametrically. n_SH_, n_EK_, and n_EW_ = 36, 33, and 47 pens, respectively

Measure	SH	EK	EW	Statistic	*P*-value
	Mean (± SEM)	Mean (± SEM)	Mean (± SEM)		
Social play	0.042 (± 0.003)	0.040 (± 0.003)	0.0044 (± 0.002)	*F*_2,70.3_ = 0.741	0.480

	Mean (± SD)	Mean (± SD)	Mean (± SD)		
Aggression	0.001 (± 0.002)	0.001 (± 0.001)	0.001 (± 0.002)	X^2^_2_ = 1.627	0.443
Defensiveness	0.001 (± 0.001)	0.000 (± 0.001)	0.000 (± 0.001)	X^2^_2_ = 3.391	0.184
Activity	0.124 (± 0.026)	0.121 (± 0.030)	0.123 (± 0.030)	X^2^_2_ = 1.142	0.565
Resting	0.112 (± 0.195)	0.076 (± 0.024)	0.080 (± 0.026)	X^2^_2_ = 1.333	0.514
Lying awake	0.002 (± 0.002)	0.003 (± 0.002)	0.002 (± 0.002)	X^2^_2_ = 1.585	0.453
Lying awake in NB	0.016 (± 0.010)	0.016 (± 0.010)	0.015 (± 0.009)	X^2^_2_ = 0.159	0.923
Inactive in NB	0.169 (± 0.041)	0.161 (± 0.037)	0.186 (± 0.162)	X^2^_2_ = 1.173	0.556

#### Pair housing

Social play differed significantly across housing conditions in the pair-housing phase; EW kits were observed performing social play more often on average than EK kits (*t*_122_ = –2.54; *P* = 0.033; Table 3[b]), although this behaviour did not differ between EW kits and SH kits (*t*_122_ = –1.11; *P* = 0.508) or between SH and EK kits (*t*_122_ = –1.52; *P* = 0.285; Table 3[b]). Aggressive and defensive behaviours remained similar across housing conditions in pair housing, as did activity levels, resting, and lying awake in the nest-box (statistics summarised in Table 3[b]). However, lying awake in the pen showed effects of housing condition towards more lying awake in EK kits than in SH kits (*W* = 3.35; *P* = 0.047); lying awake did not differ between EK and EW kits (*W* = –1.96; *P* = 0.348) or SH and EW kits (*W* = 1.87; *P* = 0.382). Inactivity in the nest-box also differed by housing condition in the pair-housing phase: EK kits were inactive in the nest-box less often on average compared to SH kits (*t*_122_ = 3.61; *P* = 0.001) and EW kits (*t*_122_ = –3.859; *P* < 0.001), while SH and EW kits did not differ in this behaviour (*t*_122_ = –0.337; *P* = 0.939; Table 3[b]).

**Table 3(b).** Effect of housing condition (standard housed, SH; enriched kits, EK; or enriched at whelping, EW) on proportions of observations where juvenile behaviours in farmed American mink (*Neogale vison*) were observed in the pair-housing period. SEM is given for measures analysed parametrically and SD is given for measures analysed non-parametrically. Significant results are in bold. n_SH_, n_EK_, and n_EW_ = 46, 37, and 42 pens, respectively

Measure	SH	EK	EW	Statistic	*P*-value
	Mean (± SEM)	Mean (± SEM)	Mean (± SEM)		
Social play	0.021 (± 0.002)	0.017 (± 0.002)	0.024 (± 0.002)	F_2,80.9_ = 3.560	**0.033**
Activity	0.231 (± 0.006)	0.242 (± 0.007)	0.239 (± 0.006)	F_2,79.2_ = 0.913	0.406
Resting	0.257 (± 0.008)	0.253 (± 0.008)	0.248 (± 0.009)	F_2,80.9_ = 0.305	0.738
Inactive in NB	0.137 (± 0.008)	0.089 (± 0.008)	0.141 (± 0.010)	F_2,80.6_ = 10.840	**< 0.001**

	Mean (± SD)	Mean (± SD)	Mean (± SD)		
Aggression	0.000 (± 0.001)	0.001 (± 0.002)	0.000 (± 0.001)	X^2^_2_ = 0.333	0.846
Defensiveness	0.000 (± 0.001)	0.001 (± 0.000)	0.000 (± 0.001)	X^2^_2_ = 1.117	0.572
Lying awake	0.001 (± 0.006)	0.014 (± 0.010)	0.011 (± 0.008)	X^2^_2_ = 6.167	**0.046**
Lying awake in NB	0.005 (± 0.006)	0.004 (± 0.005)	0.006 (± 0.007)	X^2^_2_ = 1.772	0.412

### Housing effects on kit temperament

There was no effect of housing condition on the temperaments of pair-housed kits as determined by the stick test in the summer (χ^2^_4_ = 2.31; *P* = 0.679) or fall months (χ^2^_6_ = 4.68; *P* = 0.620; Table 4). Likewise, temperaments of single-housed females did not differ across conditions in either season (χ^2^_6_ = 5.09; *P* = 0.552 and χ^2^_6_ = 3.95; *P* = 0.729, respectively; Table 4). Number of vocalisations during handling for pelt grading did not differ across conditions in pair-housed (χ^2^_2_ = 3.95; *P* = 0.832) or single-housed kits (χ^2^_2_ = 2.21; *P* = 0.332; Table 5).

**Table 4.** Summary of stick test statistics assessing temperament in pair-housed farmed American mink (*Neogale vison*) in summer (n_SH_ = 92; n_EK_ = 78; n_EW_ = 80) and fall (n_SH_ = 88; n_EK_ = 74; n_EW_ = 75) and in single-housed farmed American mink in summer (n_SH_ = 21; n_EK_ = 23; n_EW_ = 27) and fall (n_SH_ = 21; n_EK_ = 23; n_EW_ = 26) across housing conditions (standard housed, SH; enriched kits, EK; or enriched at whelping, EW). ‘S’ denotes summer test results and ‘F’ denotes fall test results

Response	Condition	Pair-housed summer stick tests				Single-housed summer stick tests			
		Response counts		% Response within condition		Response counts		% Response within condition	
		S	F	S	F	S	F	S	F
Curious	SH	66	77	71.7%	87.5%	6	10	28.6%	47.6%
	EK	58	62	74.4%	83.8%	10	14	43.5%	60.9%
	EW	52	61	65.0%	81.3%	13	14	48.1%	53.8%
Fearful	SH	9	4	9.8%	4.5%	12	1	57.1%	4.8%
	EK	7	2	9.0%	2.7%	11	2	47.8%	8.7%
	EW	12	4	15.0%	5.3%	11	2	40.7%	7.7%
Aggressive	SH	0	4	0%	4.5%	0	5	0.0%	23.8%
	EK	0	8	0%	10.8%	1	6	4.3%	26.1%
	EW	0	5	0%	6.8%	0	7	0.0%	26.9%
Unresponsive	SH	17	3	18.5%	3.4%	3	5	14.3%	23.8%
	EK	13	2	16.7%	2.7%	1	1	4.3%	4.3%
	EW	16	5	20.0%	6.7%	3	3	11.1%	11.5%

**Table 5.** Summary of pelt grading fear behaviour statistics for pair (n_SH_ = 58; n_EK_ = 60; n_EW_ = 64) and single-housed farmed American mink (*Neogale vison*) (n_SH_ = 21; n_EK_ = 23; n_EW_ = 27) across housing conditions (standard housed, SH; enriched kits, EK; or enriched at whelping, EW)

Measure	Condition	Pair-housed kits		Single-housed kits	
		Mean (± SD)		Mean (± SD)	
Vocalizations	SH	1.31 (± 6.23)		3.05 (± 6.72)	
	EK	1.30 (± 3.79)		0.74 (± 3.33)	
	EW	0.58 (± 2.58)		4.04 (± 10.2)	

	Condition	Counts (yes)	% Performance within condition	Counts (yes)	% Performance within condition
Attempts to bite the handler	SH	14	24.1%	16	76.2%
	EK	12	20.0%	13	56.5%
	EW	16	25.0%	21	77.8%
Physical struggling	SH	14	24.1%	5	23.8%
	EK	12	20.0%	6	26.1%
	EW	23	35.9%	4	14.8%
Urination	SH	2	3.4%	1	4.8%
	EK	2	3.3%	5	21.7%
	EW	1	1.6%	6	22.2%

Attempts to bite the handler occurred at similar levels between kits of each condition in pair and single housing (χ^2^_2_ = 0.490; *P* = 0.783 and χ^2^_2_ = 3.17; *P* = 0.205), as did occurrences of physical struggling (χ^2^_2_ = 4.33; *P* = 0.115 and χ^2^_2_ = 1.08; *P* = 0.582) and urination (χ^2^_2_ = 0.520; *P* = 0.742 and χ^2^_2_ = 3.13; *P* = 0.194, respectively; Table 5).

### Housing effects on kit behaviour as adults

**Figure 2.**
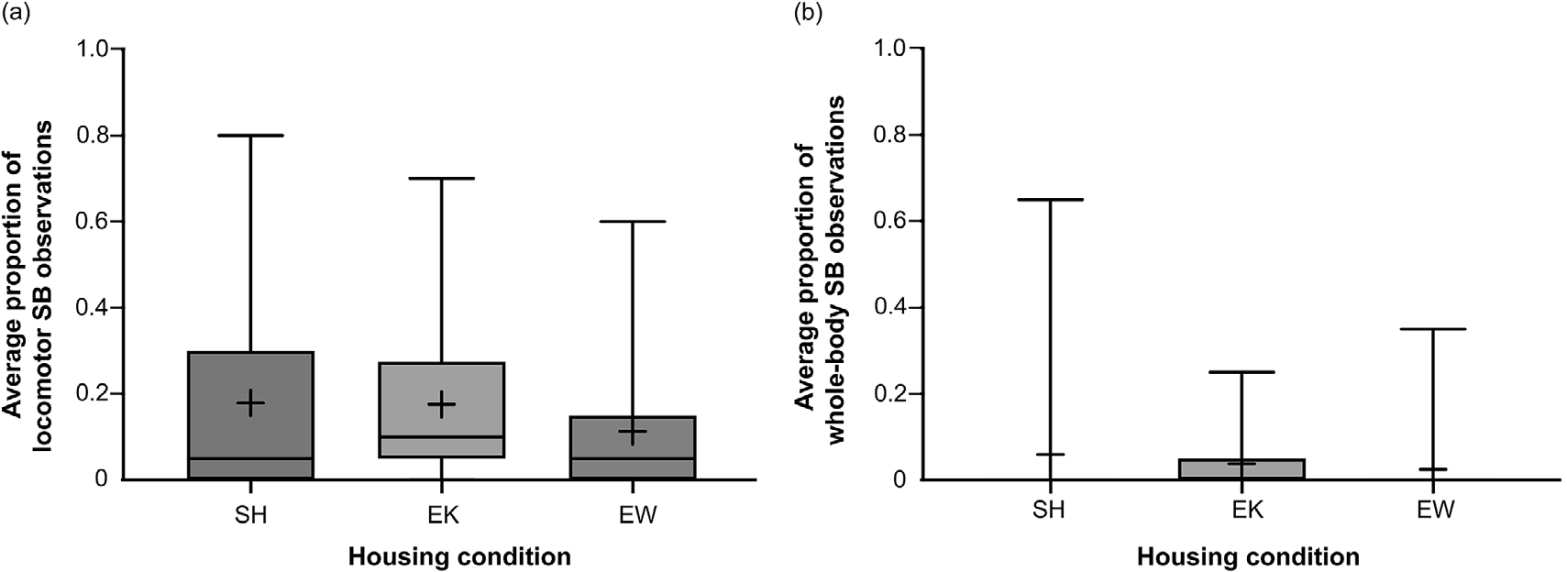
Box and whisker plots of average proportion of observations for (a) locomotor stereotypic behaviours (SBs) and (b) whole-body SBs occurring in adult farmed American mink females (*Neogale vison*) of different rearing conditions (standard housed, SH; enriched kits, EK; or enriched at whelping, EW). For each plot, n = 31, 37, and 36 sample points, respectively. Black + signs show the means.

Performance of locomotor SB and whole-body SB did not differ across adult females reared in different housing conditions (χ^2^_2_ = 3.878; *P* = 0.144 and χ^2^_2_ = 1.770; *P* = 0.413, respectively; Figure 2); there were no observations of head-based SB, scrabbling, or wire gnawing. There was also no difference in observed activity, resting, lying awake, or inactivity across housing conditions (Table 6).

**Table 6.** Effect of previous housing condition (standard housed, SH; enriched kits, EK; or enriched at whelping, EW) on behaviours of farmed American mink (*Neogale vison*) females as adults (SBs not included; presented in Figure 2). n_SH_ = 31; n_EK_ = 37; n_EW_ = 36

Measure	SH	EK	EW	Statistic	*P*-value
	Mean (± SD)	Mean (± SD)	Mean (± SD)		
Activity	0.182 (± 0.145)	0.227 (± 0.142)	0.204 (± 0.147)	X^2^_2_ = 1.856	0.395
Resting	0.485 (± 0.325)	0.446 (± 0.280)	0.556 (± 0.289)	X^2^_2_ = 2.616	0.270
Lying awake	0.050 (± 0.061)	0.043 (± 0.049)	0.040 (± 0.058)	X^2^_2_ = 0.819	0.664
Lying awake in pen^a^	0.040 (± 0.060)	0.034 (± 0.054)	0.042 (± 0.068)	X^2^_2_ = 0.171	0.918
Lying awake in NB^a^	0.008 (± 0.033)	0.007 (± 0.023)	0.002 (± 0.014)	X^2^_2_ = 0.978	0.613
Inactivity	0.042 (± 0.061)	0.066 (± 0.075)	0.061 (± 0.079)	X^2^_2_ = 2.781	0.249
Inactivity in NB^a^	0.005 (± 0.021)	0.000 (± 0.000)	0.005 (± 0.019)	X^2^_2_ = 2.311	0.315

a Locations were only recorded on three out of five days of behaviour scans.

## Discussion

As summarised in a previous article based on this study cohort, EK kits showed more overall and more sustained enrichment use across the juvenile period compared to kits in standard housing (Clark *et al.* 2023). However, as shown here, the predicted benefits of EK housing on other kit behaviours were largely unsupported. During the group-housing phase, neither positive welfare-related behaviours (social play or resting) nor behaviours potentially associated with boredom, fear, and/or stress (forms of waking inactivity and aggression) differed between SH, EK, and EW kits. During pair housing, EK kits did demonstrate reduced inactivity in the nest-box compared to SH and EW kits and increased lying awake in the pen compared to SH kits (10–13 weeks post-whelping). However, each of these forms of inactivity are suggested to be associated with negative states in mink (fear and boredom, respectively; Meagher & Mason 2012; Meagher *et al.* 2013, 2017; Fureix & Meagher 2015), and thus it is difficult to determine whether EK enrichments were able to improve kit affective states in this period. Moreover, social play occurred more often in EW kits than in EK kits during pair housing. This may be indicative of reduced stress in EW kits compared to EK kits, as social play is known to occur primarily in healthy, unstressed animals (Burghardt 2005). However, as discussed in the companion paper to this study (Clark *et al.* 2025), cortisol concentrations were not assessed during juvenile behavioural observations, and when assessed at six months of age, there was no difference in basal faecal cortisol metabolite concentrations between kits of different conditions.

The temperaments of kits reared in different conditions, as measured by the stick test, also appeared unaffected, and fear behaviours during handling for pelt grading were performed at similar levels across conditions. Occurrences of these fear behaviours were relatively low in our sample overall, as approximately twenty percent of kits demonstrated struggling, biting, or urination during handling, and a mean of only 1.05 vocalisations per kit were emitted across all conditions combined. As discussed in the companion article to this study (Clark *et al.* 2025), the mink on the research farm where experiments were conducted may have had low levels of fear as a population (fear traits are known to be highly heritable and subject to selection; Hansen 1996; Malmkvist & Hansen 2001, 2002; Berg *et al.* 2002; Thirstrup *et al.* 2019). Similarly, any effects of housing condition on fear behaviours may have been masked by individual variability in responses to handling or immobilisation stress of certain durations and intensities; individual mink have been observed to show high variation in stress responses to such events (Malmkvist *et al.* 2024). Alternatively, it is possible that the enrichment methods implemented in this study (the timing or duration of provision, rotation of items, etc) may have been ineffective in modulating mink temperament. In previous studies where minks’ temperament or fear responses to handling were improved by enriched housing, the mink were housed with enrichments consistently through to the time of assessment or temporally close to the time of assessment (Meagher *et al.* 2014; Bak & Malmkvist 2020), unlike in the present study where only a standard EE was available for all conditions at the time of assessment.

The main shortcoming of the EK intervention was that it was not successful at reducing the development of SB in the kits as adults. In fact, EK kits showed signs of developing more whole-body SB than SH or EW kits. Although there was no statistical difference in overall performance of whole-body SB, the interquartile range for this subtype in EK mink was larger than that of mink from other housing conditions. Types of SB were distinguished in the present study due to previous evidence that sub-types of SB are heterogeneous in mink, although thus far only scrabbling and head-based forms have been suggested to be distinct from locomotor forms (both stationary and those involving translocation) in their causation and treatments (Dallaire *et al.* 2011; Díez-León *et al.* 2013, 2019; Polanco *et al.* 2017, 2018). Given that locomotor forms and whole-body, stationary forms are known to co-occur in mink (Polanco *et al.* 2017; Malmkvist *et al.* 2024), the greater tendency of EK mink to display both is interesting. Overall activity also did not differ between groups, so this is an unlikely explanation for any differences in SB expression. Increases in enclosure complexity have been suggested to impede the space available to perform SB, or make them more complex in appearance (e.g. an established route-tracing stereotypy in a cape hunting dog [*Lycaon pictus*] was impeded by introduction of a hanging chain and the route was then adjusted to avoid the chain; Fentress 1976; also cf Bergeron *et al.* [2006] on oral behaviours), so it is possible that the extra mobile and hanging EEs for EK kits acted as obstacles that caused alternative, stationary forms of SB to develop.

Moreover, our finding that performance of locomotor SB or any form of SB in general was not reduced in EK mink contradicts the findings of previous studies where access to a multitude of physical enrichments in early life or adulthood positively impacted SB (Hansen *et al.* 2007; Meagher & Mason 2012; Dallaire *et al.* 2012; Campbell *et al.* 2013; Meagher *et al.* 2013; Díez-León *et al.* 2016). However, these impacts were often delivered through EE being present in the subjects’ pens at the time of SB assessment; or, in the case of Díez-León *et al.* (2016), the earliest SB assessment was conducted after only five weeks of removal from EE. The present study is the first to investigate whether providing EE to mink exclusively as juveniles can attenuate performance of SB up to one year following removal. In bank voles (*Myodes glareolus*; Ödberg 1987) and deer mice (*Peromyscus maniculatus*; Powell *et al.* 2000; Hadley *et al.* 2006), rearing in EE is shown to have protective effects against the development of SB even after long-term placement in standard pens. In other species, meanwhile, removal of temporary EE can exacerbate stereotypy (e.g. in primates: Bayne *et al.* 1992; in pigs [*Sus scrofa*]: Day *et al.* 2002; in CD-1 mice [*Mus musculus*]: Latham & Mason 2010). The latter scenario is thought to result from frustration due to placement in environments with fewer behavioural opportunities relative to their former more stimulating, rewarding environments (a so-called ‘negative contrast’ effect), whereas animals who have not experienced these enriched conditions do not have the same frustrations (Crespi 1942; Pecoraro *et al.* 1999; Wiegmann *et al.* 2003). It is therefore plausible that EK mink became accustomed to greater, more diverse enrichment and experienced frustration after subsequent placement in standard EE with only one, permanently present enrichment, resulting in development of frustration-induced SB. However, behavioural frustration in negative contrast scenarios typically also correlates with increased glucocorticoid output (Latham & Mason 2010), which was not observed in EK kits relative to kits of other conditions during faecal cortisol sampling two months after EE removal (data presented in Clark *et al.* 2025).

Moreover, in Díez-León *et al.* (2016), mink reared in EE from birth and then placed in standard housing did not demonstrate exacerbated SB following EE removal and in fact showed reductions in locomotor SB compared to mink that had never experienced enrichment. This may be due to the timing of EE removal: enriched mink were placed in standard pens for five weeks in mid-November, when mink were six or seven months of age, and their SB was assessed in these standard pens prior to them being returned to enriched pens. SB does not fully develop in mink until seven months of age (Jeppesen *et al.* 2000), at which point extra EEs for EK mink in the present study had already been removed. Thus, EE may only have protective effects against SB development in mink when supplied from rearing until approximately seven months of age, a potentially critical age in SB development. Interestingly, Axelsson *et al.* (2009) introduced enrichments to mink at seven months of age when subjects had already begun exhibiting SB, and one of the two farms housing trial mink did show an enrichment-related decrease in SB while the other did not. Thus, it is possible that seven months is a critical age for the provision of EE in mink, but provision leading up to this age is more effective than introduction once SBs have already begun to develop. In the aforementioned studies with deer mice, EE was also provided early in life (from 4–14 or 14–21 weeks of age: Hadley *et al.* 2006; detailed timing of provision is unknown in Powell *et al.* 2000), and SB in deer mice is thought to become stable by six weeks of age (Tanimura *et al.* 2010). Thus, the mice also had access to enrichments prior to and during the period in which SB becomes fully developed, and this EE strategy was also protective against future development of SB.

Of course, the lack of SB reduction in EK mink, or lack of statistical difference in whole-body SB of mink across housing conditions, may also have resulted from a false negative. Our sample of mink followed for SB development as adults was limited due to pelting of select individuals in their first year of life (according to standard farm practices) and other factors such as mortality, thus affecting our statistical power to detect differences. In future research it would be beneficial to follow a larger sample of females (and potentially males, as well) through to adulthood to observe their SB development after differential housing as juveniles. Alternatively, the amount of behavioural data collected from the available mink could be increased (i.e. observations could be conducted across a longer period than five days, or the number of scans per day could be increased). Regarding our speculations about timing of removal for EK enrichments, we recommend that future studies attempt to house mink with the nature of EE provided in this study until seven months of age to determine if this duration and timing of juvenile enrichment is sufficient to deliver lasting preventative effects on SB, even after long-term placement in standard housing.

### Animal welfare implications

Although used, the manipulable play objects provided to enriched mink kits in this study did not deliver many positive impacts on behavioural measures of welfare. These enrichments may have the potential to reduce stereotypic behaviour development if access is maintained beyond 15 weeks of age. The results of this study provide a basis for the hypothesis that environmental enrichment may be more effective at modulating stereotypic behaviour if provided during critical periods of stereotypic behaviour development, which may have applications for welfare intervention strategies in various species that tend to perform locomotor and whole-body forms of stereotypic behaviour in captivity.

## Conclusion

Providing farmed mink kits with an array of manipulable EEs periodically exchanged to maintain novelty was successful at reducing a potential behavioural indicator of fear in the late juvenile period (inactivity in the nest-box), but a potential indicator of boredom was simultaneously increased (lying awake in the pen). Stereotypic behaviour development in the kits as adults was also not affected by EK housing; this demonstrates that additional enrichment as juveniles may not attenuate stereotypic behaviour performance in adult mink after one year of placement in standard housing. It would be valuable to determine in future studies if juvenile enrichment of this nature has long-term benefits when maintained until seven months of age, when stereotypic behaviours are known to become established in mink.
